# Beware the myth: learning styles affect parents’, children’s, and teachers’ thinking about children’s academic potential

**DOI:** 10.1038/s41539-023-00190-x

**Published:** 2023-10-17

**Authors:** Xin Sun, Owen Norton, Shaylene E. Nancekivell

**Affiliations:** 1 University of British Columbia, Vancouver, BC Canada; 2 University of North Carolina Greensboro, Greensboro, NC USA; 3 University of Manitoba, Winnipeg, MB Canada

**Keywords:** Human behaviour, Education, Education

## Abstract

Three experiments examine how providing learning style information (a student learns hands-on or visually) might influence thinking about that student’s academic potential. Samples were American and predominately white and middle-class. In Experiment 1, parents (*N* = 94) and children (*N* = 73, 6–12 years) judged students who learn visually as more intelligent than hands-on learners. Experiment 2 replicated this pattern with parents and teachers (*N* = 172). In Experiment 3 (pre-registered), parents and teachers (*N* = 200) predicted that visual learners are more skilled than hands-on learners at “core” school subjects (math/language/social sciences, except science), whereas, hands-on learners were skilled at non-core subjects (gym/music/art). Together, these studies show that learning style descriptions, resultant of a myth, impact thinking about children’s intellectual aptitudes.

## Introduction

The learning style myth is widely endorsed by educators and the general public across countries, including those in the United States, Turkey, Portugal, China, Switzerland, the United Kingdom, and Latin America^1–11^. The most common, but erroneous, models of learning styles focus on children as possessing a dominant way of learning tied to a singular learning modality, such as a visual, auditory, or kinesthetic/tactile learning style (i.e., the VAK model^1,12^). Despite its popularity, there is no substantial evidence to support the idea that people have distinct dominant styles of learning that are tied to a singular modality^13–16^. For example, there is no evidence that VAK learning styles predict cognitive functioning or learning. VAK learning styles are unrelated to the evidence-based ways that researchers have successfully characterized individual differences in thinking^17–20^.

The present study investigates an overlooked, but likely serious consequence of the learning style myth: how learning style information may influence parents’, teachers’ and children’s thinking about young students’ potential. Scientists have long argued that the main detriment of the learning style myth is that it leads educators to waste resources on ineffective methods that could be spent on evidence-based ones^13–16^. However, the pervasiveness of the myth makes it likely that its consequences extend beyond solely wasted resources. The current study addresses this issue by examining whether teachers, parents, and children think those described as having certain learning styles are smarter than others (i.e., learning better with one’s hands as a hands-on vs. learning better with one’s eyes as a visual learner), and similarly, how learning style information affects teachers’ and parents’ thinking about young children’s ability to excel in different school subjects.

Prior work suggests that embedded in the (VAK) learning style myth may indeed be erroneous beliefs about young students’ educational abilities. There is also a large body of work treating learning styles as real phenomena, which has sought to uncover “field-specific” beliefs about learning styles and their link to academic success (e.g., STEM learning; medicine^21,22^). Inherent in this work is a similar suggestion that certain learning styles may be more common and/or more likely to lead to success than others in certain academic fields. For example, one study erroneously linked visual learning style with academic success in applied science courses^23^ while another study concluded that medical students have a different learning style than the general population^21^. Moreover, these kinds of links have been spread across university settings with staff from teaching centers lecturing on how auditory learners “*flourish in foreign language learning*”, and “*chemists and engineers are often kinesthetic learners*”^24^. Together, prior work suggests that it is likely that supposed learning styles are viewed as informative about children’s intelligence, and their success in specific academic domains (art vs. science).

Several recent empirical works probing learning styles beliefs provide theoretical support for the proposal that people link learning styles to student potential. They specifically suggest that many people view learning styles through an essentialist lens^6,25^. Psychological essentialism is a belief that a category has “true nature” that is based in biology, and which determines the behavior of its category members^26,27^. In the case of learning styles, this work showed that people believe that learning in a kinesthetic, auditory, or visual manner are markers of stable categories (e.g., visual or kinesthetic/hands-on learning styles) that are based in the biology of the brain, and predict life and educational outcomes^6^. A hallmark of such essentialist reasoning is that it facilitates specific inductive generalizations about behavior^28^. For example, in the case of gender, essentialist reasoning leads children to believe that a girl, by virtue of being a girl, will play with dolls, and wear dresses^29^. In the case of learning styles, prior work has only examined whether people generally agree that their perceived learning styles predict learning outcomes, and not how they predict specific behaviors or academic performance. In other words, prior work has not examined the kinds of predictions or inductive inferences about academic performance that learning styles might lead to. The present study seeks to answer this question by examining the specific inferences that learning styles lead parents, teachers, and children to make about young students’ educational outcomes and potential (i.e., how ‘smart’ a child is, or good at math).

The possibility that people may believe that perceived learning styles are predictive of student potential is concerning. From childhood, people’s theories of learning are complex and have consequences on their thinking about themselves and other people^30–32^. By first grade, children hold implicit theories of intelligence^33^, and many view intelligence as a stable fixed trait^32^. Young children also hold stereotypes about intelligence. For example, 6-year-olds have gendered notions of brilliance wherein they believe that boys are more likely than girls to be “really really smart”^30^, and they similarly think that boys are better at math than girls^34,35^. At this age, racial stereotypes also influence children’s thinking about achievement: They associate white men, but not Black men with brilliance^36^. In adulthood, such stereotypes remain. They additionally influence thinking about who is likely to be smart, and participate in STEM fields^37,38^.

Further, such beliefs have ramifications for children: They affect their educational preferences and engagement in science^30,34,35,38^. They influence children’s perceptions of classmates, thereby impacting peer acceptance and classroom engagement^39^. In adulthood, stereotypes related to brilliance are related to the distribution of women in scientific fields, and to experiences of imposter syndrome^37,40^. Parents’ beliefs about their child’s math abilities are related to their child’s math achievement^35^. Educators’ beliefs about whether intelligence is fixed at birth are related to their students’ achievement, especially among stigmatized groups^41^. Taken together, these bodies of work demonstrate that people’s beliefs about learning and intelligence emerge early and have measurable real-life consequences.

### The Present Studies

Here, we conduct three experiments which are the first to test whether (and how) learning style information influences thinking about children’s academic potential. Throughout these experiments, we rely on descriptions (e.g., “a student who learns best by using their hands”) instead of only labels (e.g., “hands-on learner”) to account for potential differences in label familiarity among children, parents, and teachers (e.g., tactile, hands-on, and kinesthetic are all similar labels). Moreover, the reliance on descriptors is ecologically valid as such descriptors are always provided in learning style assessments and reports^42,43^. We modeled our descriptors closely after such real-life discussions (see Supplementary Table 1 for descriptions from online blogs and learning styles assessment tools).

In Experiment 1, children and parents were introduced to two young students who were described as a visual or hands-on learner. They were then asked to rate their intelligence and sportiness (athleticism). We include children as one of the samples to explore whether they may embrace the idea of learning styles and how they may reason about learning styles. In Experiment 2, parents and teachers were asked to select which learner was smarter in a forced-choice scenario. In both these experiments, we compare thinking about intelligence to athleticism/sportiness to ensure findings are not the result of a global halo effect wherein one kind of learner is indiscriminately rated higher. Experiment 2 predicted that the effect we found in Experiment 1 would be amplified in this forced-choice ranking scenario. Children were not tested in Experiment 1 or subsequent studies due to their limited availability during COVID-19 pandemic. To dive deeper into the perceived weaknesses and strengths of each learner, in Experiment 3 (pre-registered), parents and teachers predicted the report card grades of hands-on and visual learners across common elementary school subjects. As many real-world discussions (i.e., online educational blogs) often mistakenly associate hands-on learners with sports, arts, and visual learners with core school curriculum (e.g., reading^44,45^), we hypothesized that the visual learner would be rated as more intelligent and competent at core school subjects (i.e., math, language arts, science, and social studies) whereas the hands-on learner would be rated as more sporty and competent at non-core school subjects (i.e., arts, gym, and music). The predicted link between aptitude (intelligence) and core subjects was driven by both real word discussions (see review above) and by prior work examining which fields are thought to require brilliance^30^. The data, analytic code, and materials for Experiments 1 to 3 are publicly accessible: Link. Experiment 3 is preregistered at: Link.

## Results

### Experiment 1

#### Analytic Plan

Parents’ and children’s answers were scored using a four-point scale as follows: 1 as not smart or sporty, 2 as sort of smart or sporty, 3 as smart or sporty, and 4 as really smart or sporty. Next, linear mixed-effects regression models were built using R package lme4. Cohen’s *d* was calculated for effect sizes. 95% Confidence intervals were calculated using Wald method. In all models, random effects were modeled for each participant as (1|id). Post hoc simple effects tests were conducted with R package emmeans and *p*-values were Bonferroni corrected. For critical effects, post hoc sensitivity analyses were conducted using the R package simr. Children and adults participated in slightly different variants of the study, and data were analyzed separately.

#### Children’s responses

We first fit an omnibus mixed linear effects model including main effects of Age (centered, continuous in months), Learning Style (visual, hands-on), and Question Type (smart, sporty) and all interaction terms to test which factors predicted participant ratings. There was a significant Learning Style main effect, *b*_LearningStyle_ = 0.22, 95% CI = [0.09, 0.34], *p* < 0.001, *d* = 0.47. The interaction between Question Type and Learning Style was also significant, *b*_Learning Style*Question_ = −0.20, 95% CI = [−0.32,−0.08], *p* = 0.001, *d* = −0.44. A post hoc sensitivity analysis determined that the sample was adequately powered to detect this main interaction, 90.30% power with a 95% CI = [88.30%, 92.06%]. All the remaining terms were non-significant.

**Fig. 1:**
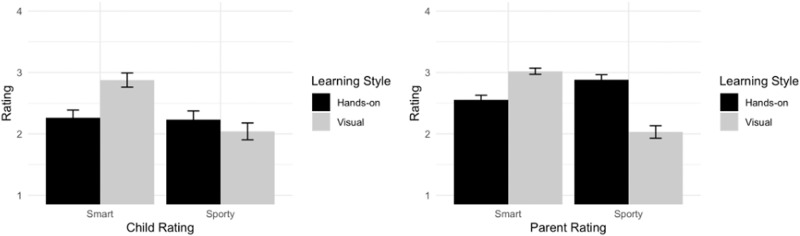
Study 1 smart and sporty ratings (Mean and Error bars showing s.e.m) by learning style. Left panel: ratings from the child sample. Right panel: ratings from the parent sample. Ratings were converted to 1-4 to indicate how smart/sporty the participants think about the student described as a visual/hands-on learner. 1 - not very smart/sporty, 2 - sort of smart/sporty, 3 - smart/sporty, 4 - really smart/sporty.

We next decompose the significant two-way Question Type by Learning Style interaction using follow-up simple effects contrasts (Bonferroni corrected for two tests). Results showed that children viewed visual learners (*M(SD)*_Visual_ = 2.88 (0.99)) as significantly smarter than hands-on learners (*M(SD)*_Hands-on_ = 2.26 (1.09), Fig. 1 left panel), *t* = 3.48, *p*_adjusted_ = 0.001, *d* = 0.58. In contrast, children rated visual and hands-on learners (*M(SD)*_Visual_ = 2.04 (1.17)) as similarly sporty (*M(SD)*_Hands-on_ = 2.23 (1.21), Fig. 1 left panel), *t* = 1.08, *p* = 0.540, *d* = 0.18. Scatterplots, provided in our supplementary materials, illustrate the absence of an age effect. These graphs plot each Question Type and Learning Style and are presented in Supplementary Fig. 1.

#### Parents’ responses

A similar set of analyses were then conducted with the parent sample but without age. Results showed that all main effects and interactions were significant. For the main effect of Question Type, *b*_Question_ = 0.16, 95% CI = [0.10, 0.23], *p* < 0.001, *d* = 0.56. For the main effect of Learning Style, *b*_Learning Style_ = 0.10, 95% CI = [0.03, 0.17], *p* = 0.007, *d* = 0.32. For the interaction between Learning Style and Question Type, *b*_Learning Style*Question_ =–0.33, 95% CI = [–0.40, –0.26], *p* < 0.001, *d* = –1.11. A post hoc sensitivity analysis determined that the sample was adequately powered to detect this main interaction, 100% power with a 95% CI (99.63%, 100%).

To parse out the interaction effect, we again conducted follow-up simple effects contrasts (Bonferroni corrected for two tests). Results showed that parents rated visual learners (*M(SD)*_Visual_ = 3.02 (0.49)) as significantly smarter than hands-on learners (*M(SD)*_Hands-on_ = 2.55 (0.74), Fig. 1right panel), *t* = 4.66, *p*_adjusted_ < 0.001, *d* = 0.68. In contrast, parents viewed hands-on learners (*M(SD)*_Hands-on_ = 2.88 (0.79)) as significantly sportier than visual learners (*M(SD)*_Visual_ = 2.03 (0.99), Fig. 1 right panel), *t* = 8.47, *p*_adjusted_ < 0.001, *d* = 1.24.

### Experiment 2

We fit a mixed binary logistic regression to examine whether question (smarter and sportier) and group membership (teacher and parent) predicted participants’ selections (the visual or hands-on learner). Identical to Experiment 1, random effects were again modeled for each participant. The same packages were used as in Experiment 1. The main difference was that due to the binary nature of the DV, odds ratios are reported instead of Cohen’s d.

**Fig. 2:**
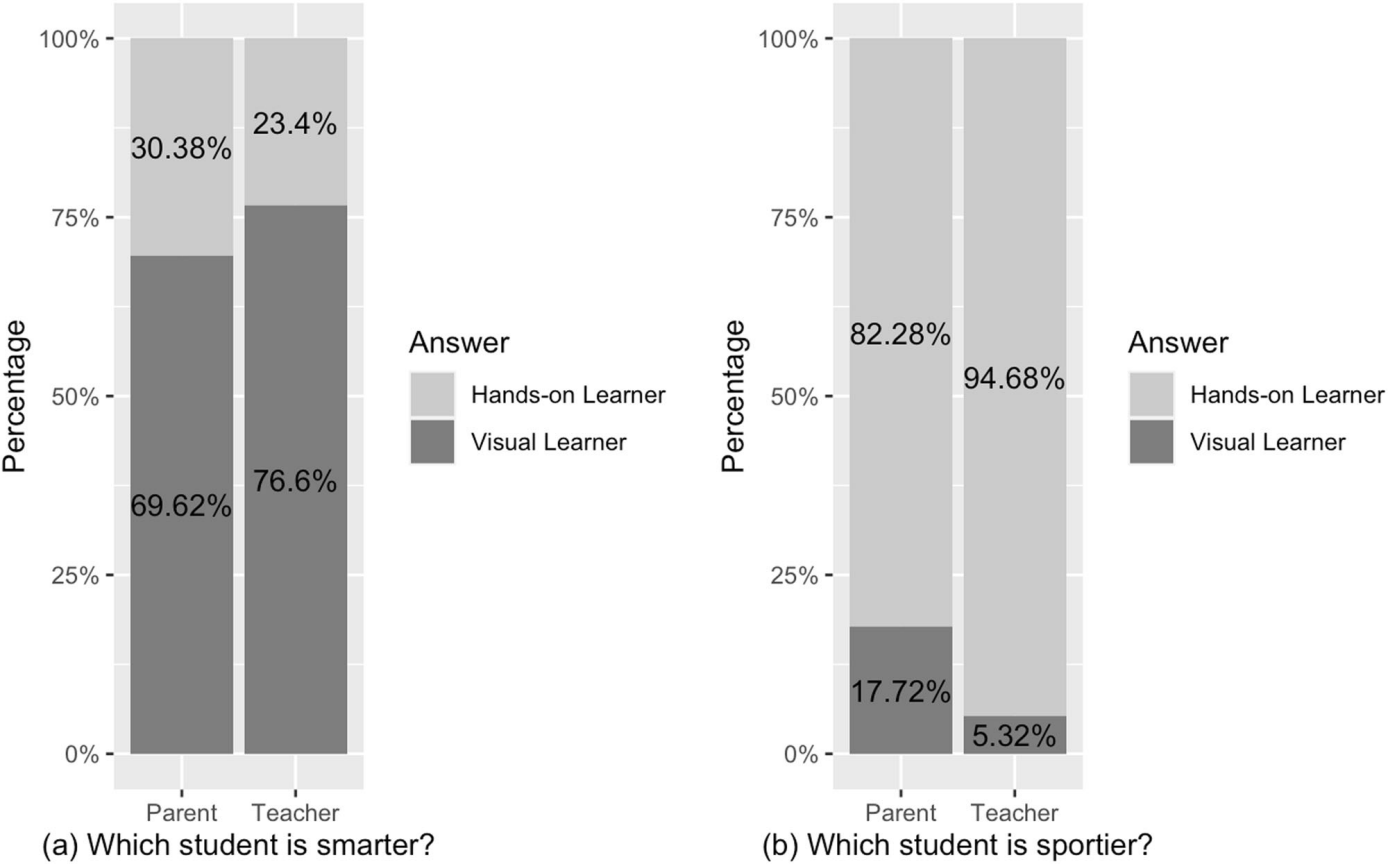
Study 2 participants’ selections of each student type (percentage) by question and participant group. **a** percentage to the question “Which student is smarter”. **b** percentage to the question “Which student is sportier” Each bar indicates the percentage that the sample selected the visual or hands-on learner as “smarter” or “sportier”, adding up to 100%.

Figure 2 displays the percentage of participants’ answers who selected each student type (visual or hands-on learner) divided by question (smarter and sportier) and participant group (teacher and parent). The main effect of the question type was significant, *b* = 1.61, 95% CI = [1.29, 1.92], *z* = 9.95, *p* < 0.001, odds ratio = 4.99. There was no main effect of group on judgments (parent or teacher), *b* = 0.25, 95% CI = [–0.07, 0.56], *z* = 1.53, *p* = 0.126, odds ratio = 1.28. But there was a significant interaction between the question and group, *b* = –0.43, 95% CI = [–0.74, –0.11], *z* = –2.63, *p* = 0.008, odds ratio = 0.65. As shown in Fig. 2, participants were more likely to characterize the visual learner as smarter than the hands-on learner, but the hands-on learner as sportier than the visual learner. In terms of interaction, teachers showed a larger effect than parents (again see Fig. 2).

**Fig. 3:**
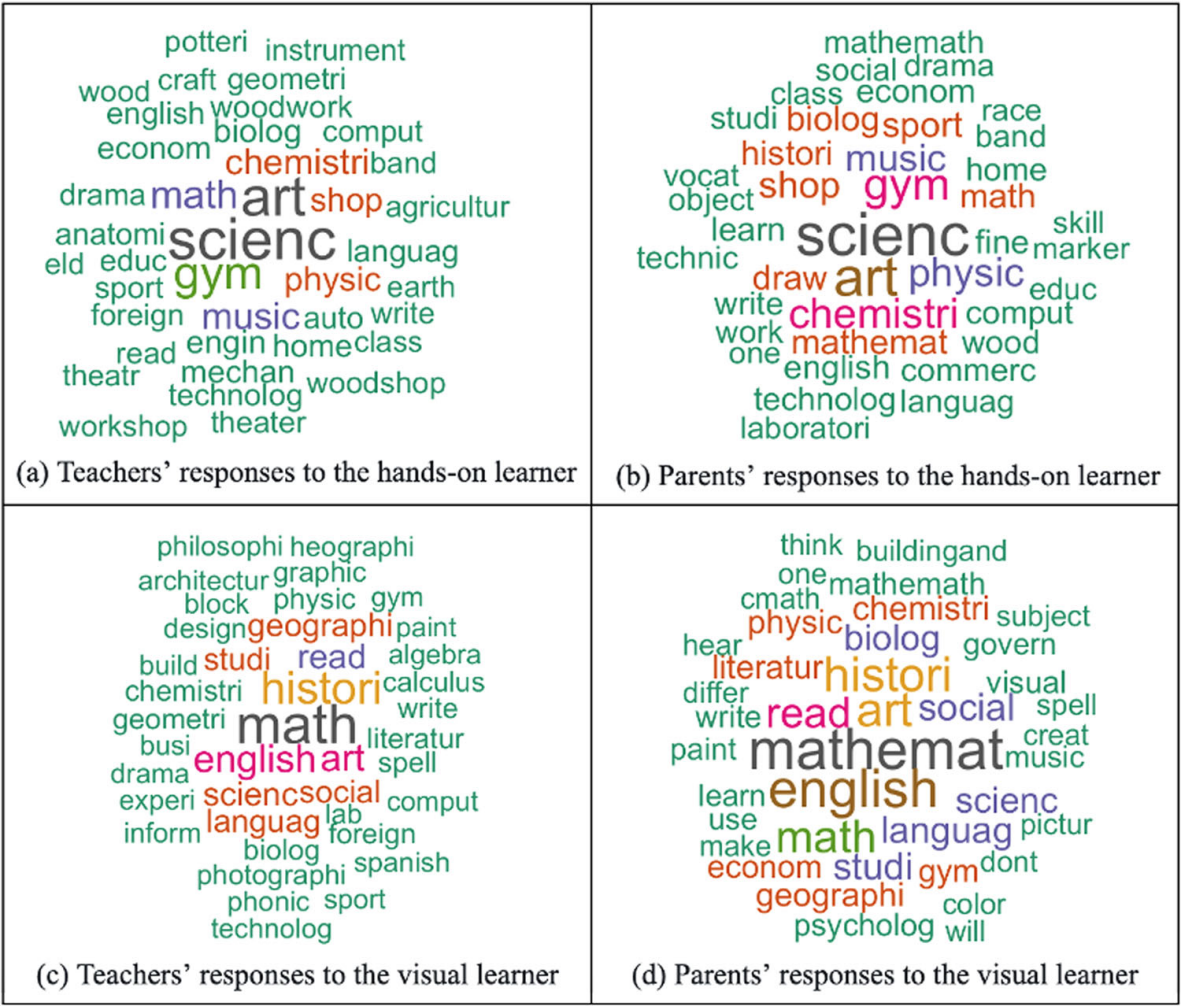
Participants’ responses to the open-ended questions, divided by question and participant group. Color and word size indicate the frequency of the word (word stems used).

For the exploratory open-ended question, we then drew word clouds for each learner style (Fig. 3) to help us to visualize the frequency with which different subjects were listed. As shown in Fig. 3 parents and teachers viewed each learner as having different educational strengths. Next, we break down those strengths using frequency data.

#### Visual learner

Teachers viewed visual learners as likely to excel at math/mathematics (22.65%), history (14.89%), English (9.39%), art (8.74%), and reading (5.83%). Similarly, the top five subjects listed by parents were math/mathematics (19.56%), English (9.96%), art (7.75%), history (7.75%), and reading (5.54%).

#### Hands-on learner

Teachers viewed hands-on learners as likely to excel in science (19.14%), art (16.83%), gym (11.55%), math (6.60%), and music (5.28%). Similarly, the top five subjects listed by parents were science (13.52%), art (11.03%), gym (6.76%), chemistry (5.34%), and physics (4.63%). We note that collapsing across subcategories of science (e.g., chemistry, physics) does not change the nature of the top three subjects.

### Experiment 3

The same packages and analytic method were used as in Experiment 1. However, we did not use Bonferroni correction because of pre-registration.

#### Pre-registered Hypothesis 1

##### Hypothesis

Visual learners will be rated as having higher grades than hands-on learners for “traditional” core school subjects (e.g., math, science, social studies, language arts). In contrast, we predict that hands-on learners will be rated higher on non-core subjects than visual learners (e.g., art, music, gym).

##### Analysis

We ran a mixed linear effects model including main effects of Subject Type (core, non-core), Learning Style (visual, hands-on), and Sample (teacher, parent) and all their interaction terms to predict grade ratings. There were significant main effects of Learning Style, *b*_Learning Style_ = 0.07, 95% CI = [0.004, 0.128], *p* = 0.036, *d* = 0.08, and Subject Type, *b*_Subject Type_ = –0.69, 95% CI = [–0.75, –0.63], *p* < 0.001, *d* = –0.86, but not Sample, *b*_Sample_ = –0.05, 95% CI = [–0.17, 0.08], *p* = 0.456, *d* = –0.11. There was a significant interaction between Learning Style and Subject Type, *b*_Learning Style*Subject Type_ = –0.60, 95% CI = [–0.66, –0.54], *p* < 0.001, *d* = –0.75. A post hoc sensitivity analysis determined that the sample was adequately powered to detect this interaction, 100% power with a 95% CI (99.63%, 100%).

**Fig. 4:**
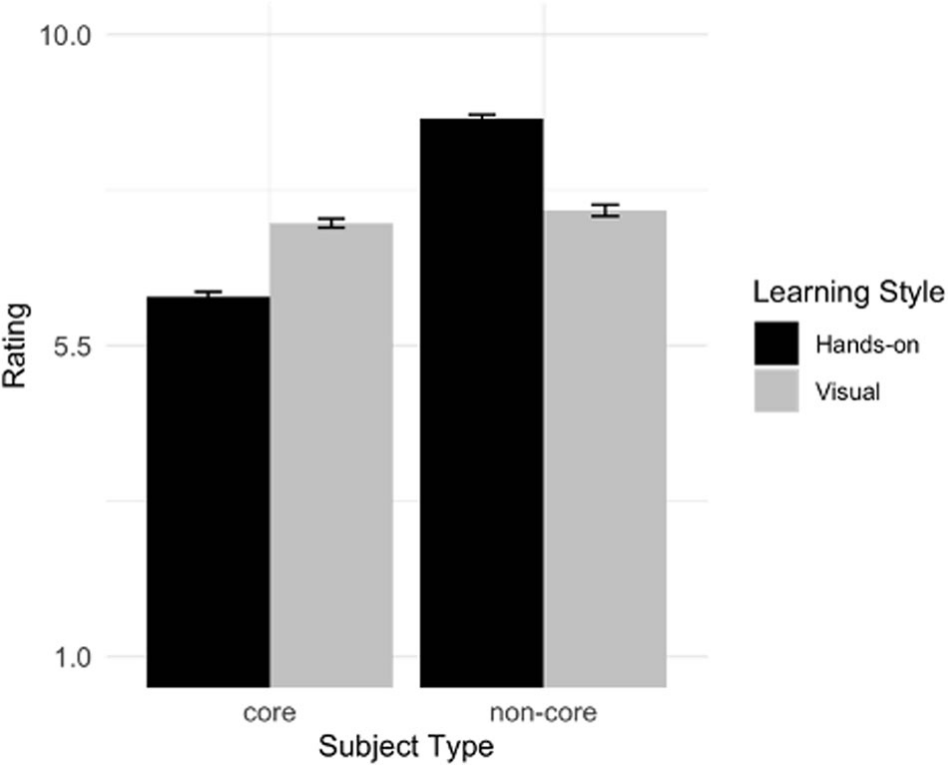
Study 3 participant grade ratings (Mean and Error bars showing s.e.m) by learning style and subject type. Grade ratings were on a 1–10 scale, representing letter grades from Below C-, C-, C, C+,… A+, respectively. Each bar indicates the mean grade rating with error bars showing s.e.m.s. Subject type is divided into core (math, science, language arts, social studies) and non-core (art, gym, music).

We next decomposed the significant two-way Learning Style by Subject Type interaction using simple effects contrasts. As predicted, for core subjects, participants rated the visual learner as having higher grades than the hands-on learner, *t* = 12.89, *p* < 0.001, *d* = 0.65. In contrast, for non-core subjects, participants rated the hands-on learner as having higher grades than the visual learner, *t* = 13.94, *p* < 0.001, *d* = 0.81 (Fig. 4).

#### Pre-registered Hypothesis 2 A

##### Hypothesis

Visual learners will be rated as having higher grades for math, social studies, and language arts, but not science. For science, we predict an opposite trend wherein hands-on are rated as having higher grades than visual learners.

##### Analysis

We ran a mixed linear effects model which tested whether the main effects of Subject (math, social studies, language arts, science), Learning Style (visual, hands-on), and their interaction predicted grade ratings. There were significant main effects of Learning Style, *b*_Learning Style_ = –0.53, 95% CI = [–0.60, –0.46], *p* < 0.001, *d* = –0.79, and Subject, *b*_Subject_ = 0.97, 95% CI = [0.85, 1.09], *p* < 0.001, *d* = 0.83. The interaction between Learning Style and Subject also yielded significance, *b*_Learning Style*Subject_ = 0.62, 95% CI = [0.50, 0.74], *p* < 0.001, *d* = 0.53. A post hoc sensitivity analysis determined that the sample was adequately powered to detect this interaction, 100% power with a 95% CI (99.63%, 100%).

**Fig. 5:**
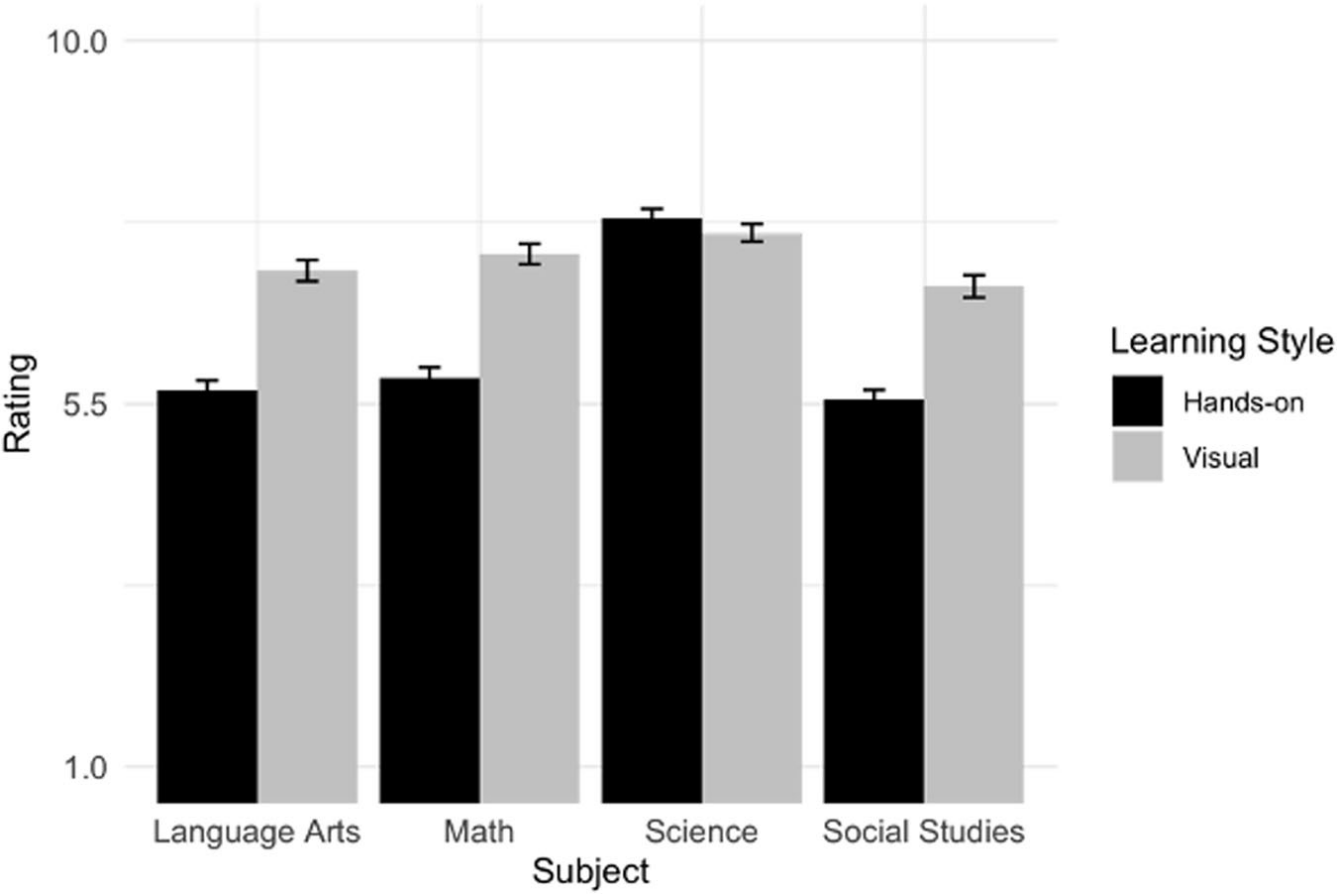
Study 3 participant grade ratings (Mean and Error bars showing s.e.m) by learning style and core subject. Grade ratings were on a 1–10 scale, representing letter grades from Below C-, C-, C, C+,… A+, respectively. Each bar indicates the mean grade rating with error bars showing s.e.m.

We next decompose the significant two-way Learning Style by Subject interaction using simple effects contrasts. As predicted, participants rated the visual learner to score higher than the hands-on learner in language arts ((*M(SD)*_Visual_ = 7.15(1.86), (*M(SD)*_Hands-on_ = 5.67(1.69), *t* = 10.23, *p* < 0.001, *d* = 1.02), math ((*M(SD)*_Visual_ = 7.36(1.79), (*M(SD)*_Hands-on_ = 5.82(1.91), *t* = 10.65, *p* < 0.001, *d* = 1.07), social studies ((*M(SD)*_Visual_ = 6.96(1.96), (*M(SD)*_Hands-on_ = 5.55(1.72), *t* = 9.72, *p* < 0.001, *d* = 0.97), but not science (*M(SD)*_Visual_ = 7.62(1.55), (*M(SD)*_Hands-on_ = 7.80(1.72), *t* = 1.21, *p* = 0.226, *d* = 0.12, Fig. 5).

#### Pre-registered Hypothesis 2B

##### Hypothesis

Hands-on learners will be rated higher on all non-core subjects than visual learners.

##### Analysis

Similar to the analysis of Hypothesis 2 A, we first ran an omnibus analysis which examined whether the main effects of Subject (art, gym, music) and Learning Style (visual, hands-on) and their interaction predicted grade ratings. There were significant main effects of Learning Style, *b*_Learning Style_ = 0.66, 95% CI = [0.59, 0.74], *p* < 0.001, *d* = 1.06, and Subject, *b*_Subject_ = 0.73, 95% CI = [0.62, 0.84], *p* < 0.001, *d* = 0.83. The interaction between Learning Style and Subject was also significant, *b*_Learning Style*Subject_ = –0.43, 95% CI = [–0.54, –0.32], *p* < 0.001, *d* = –0.48. A post hoc sensitivity analysis determined that the sample was adequately powered to detect this interaction, 100% power with a 95% CI (99.63%, 100%).

**Fig. 6:**
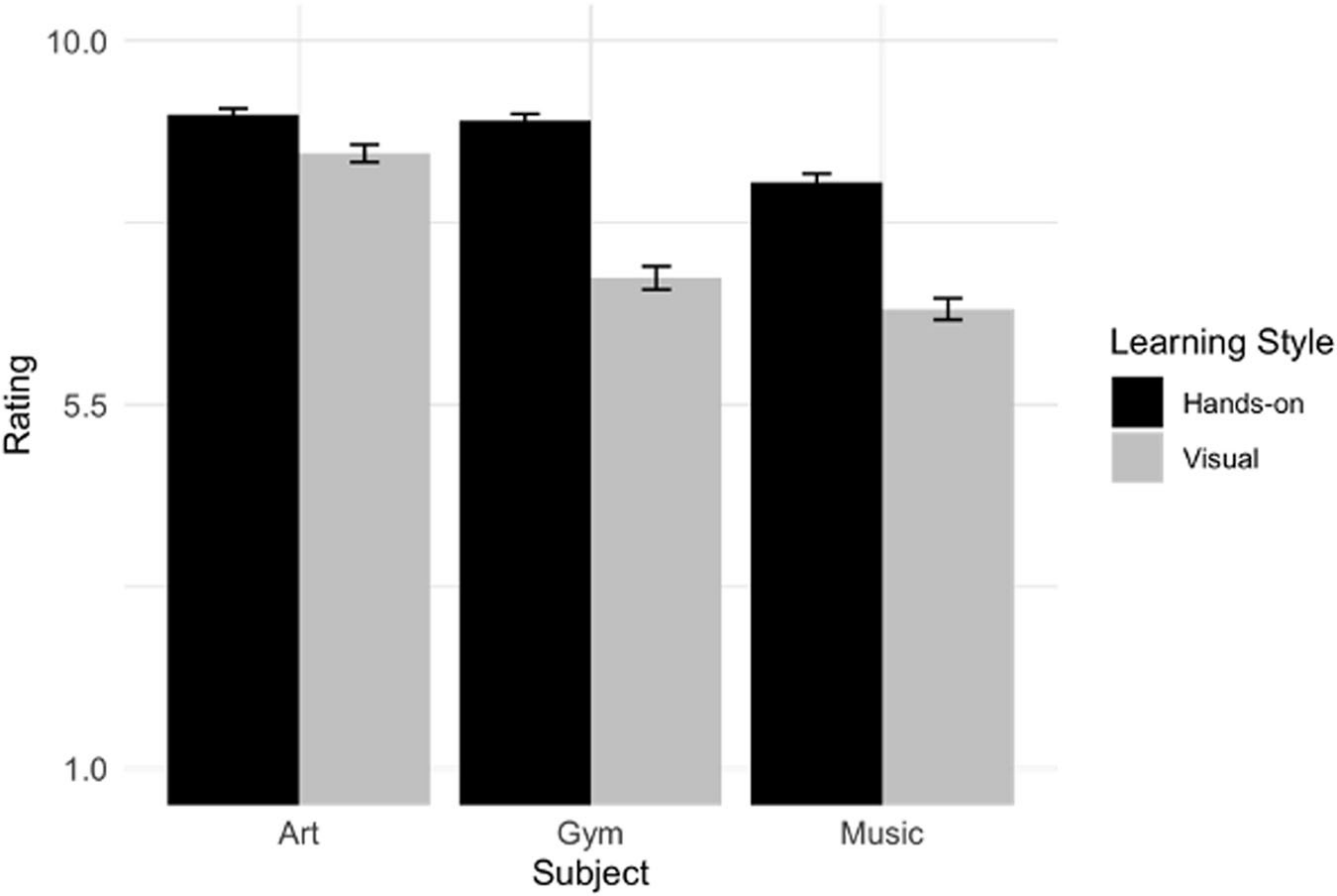
Study 3 participant grade ratings (Mean and Error bars showing s.e.m) by learning style and non-core subject. Grade ratings were on a 1–10 scale, representing letter grades from Below C-, C-, C, C+,… A+, respectively. Each bar indicates the mean grade rating with error bars showing s.e.m.

We next decomposed the significant two-way Learning Style by Subject interaction using simple effects contrast tests. As predicted, participants rated the hands-on learner to score higher than the visual learner in Art ((*M(SD)*_Visual_ = 8.61(1.55), (*M(SD)*_Hands-on_ = 9.09(1.10), *t* = 3.47, *p* < 0.001, *d* = 0.35), Gym ((*M(SD)*_Visual_ = 7.07(2.04), (*M(SD)*_Hands-on_ = 9.01(1.23), *t* = 14.16, *p* < 0.001, *d* = 1.42), and Music ((*M(SD)*_Visual_ = 6.69(1.87), (*M(SD)*_Hands-on_ = 8.25(1.56), *t* = 11.42, *p* < 0.001, *d* = 1.14, Fig. 6).

#### Pre-registered Hypothesis 3

##### Hypothesis/goal

Replication of Experiment 2 findings that visual learners are perceived to be smarter than hands-on learners, and that teachers show a larger effect.

##### Analysis

**Fig. 7:**
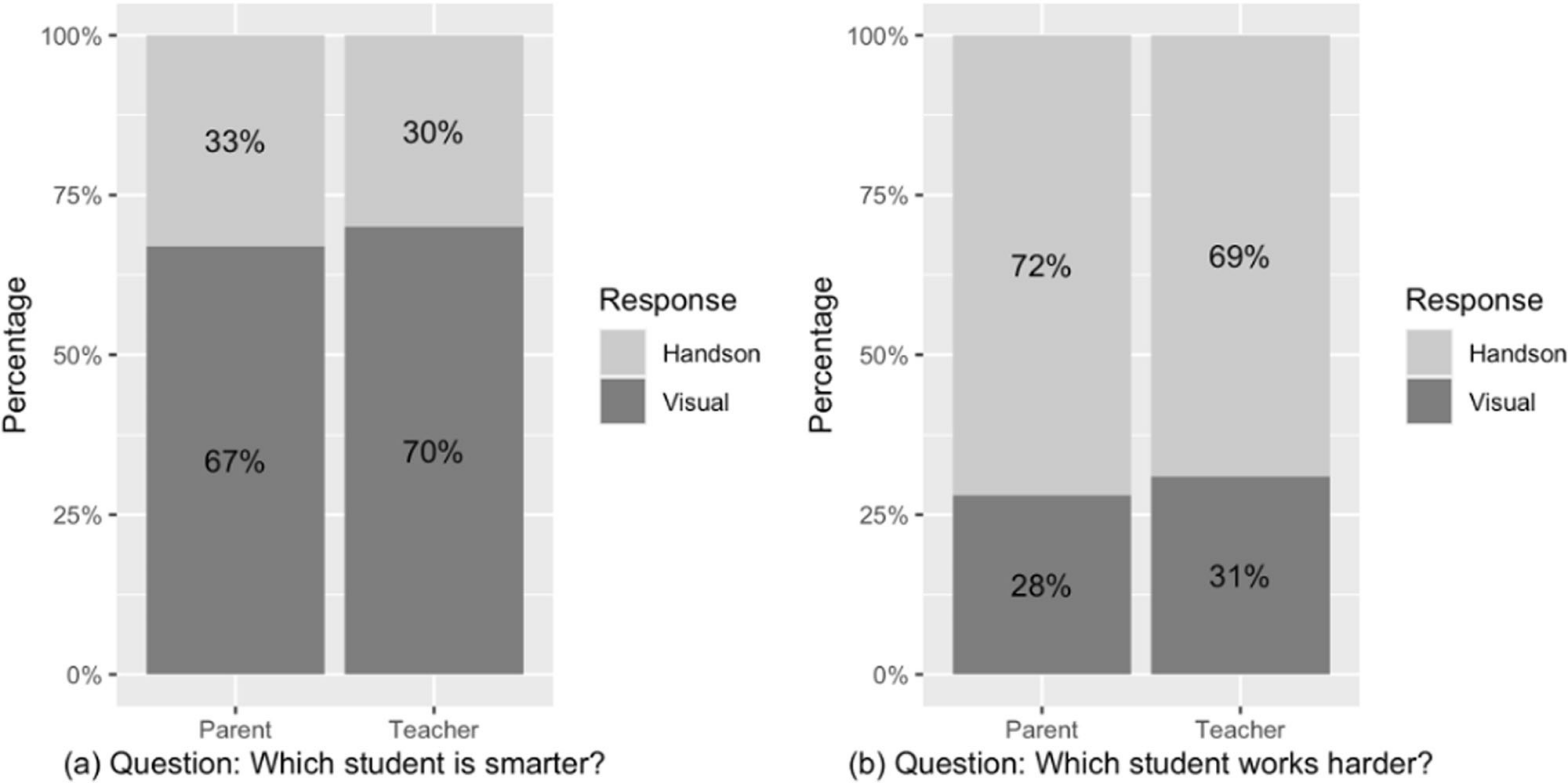
Study 3 participants’ selections of each student type (percentage) by question and participant group. **a** percentage to the question “Which student is smarter”. **b** percentage to the question “Which student is sportier”. Each bar indicates the percentage that the sample selected the visual or hands-on learner as “smarter” or “sportier”, adding up to 100%.

Figure 7 displayed the percentage of participants’ answers of student type (visual/hands-on learner) by question type (smarter and harder) and sample group (teacher and parent). We fit a mixed effects binary logistic regression to examine whether the question (smarter, works harder) and group (teacher, parent) predicted participants’ answers (the visual or hands-on learner). The main effect of the question type was significant, *b* = –0.83, 95% CI = [–1.04, –0.612], *z* = –7.59, *p* < 0.001, odds ratio = 0.44. Participants were more likely to pick the visual learner as smarter than the hands-on learner; in contrast (Fig. 7a), they were more likely to pick the hands-on learner as working harder than the visual learner (Fig. 7b). There was no main effect of the sample group (parent or teacher), *b* = –0.071, 95% CI = [–0.28, 0.14], *z* =–0.65, *p* =0.515, odds ratio = 0.93. The interaction between the question and group was not significant, *b* = –0.001, 95% CI = [–0.21, 0.21], *z* = –0.01, *p* = 0.990, odds ratio = 1.00. We did not replicate the differences between parents and teachers from Experiment 2 and so it is not discussed further.

## Discussion

Parents, teachers, and children judged children described as visual learners as more intelligent than children described as hands-on learners. Compatible with these judgments, teachers and parents also predicted that children described as visual learners would receive higher grades than those described as hands-on learners in the majority of “core” school subjects, including math, social sciences, and language arts. In contrast, children described as hands-on learners were viewed as being more skilled at non-core subjects including, gym, music, and with smaller effects, art. The exception to this pattern was science wherein children described as hands-on learners were viewed as equally skilled at (elementary school) science. Together, these findings reveal a new consequence of the learning style myth: Perceived learning styles influence people’s thinking about young students’ academic potential and abilities. Describing students as being hands-on or visual learners influenced parents’ and teachers’ thinking about those young students’ intelligence and academic achievement (i.e., report card grades).

The present study is also the first to investigate young children’s (6–12 years) beliefs about the learning style myth (Experiment 1). We provide evidence that learning style information likely influences children’s thinking. Namely, describing peers as hands-on or visual learners influenced some children’s judgments of their peers’ intelligence and athleticism. Together, these findings are the first to suggest that any neuromyth influences elementary school children’s thinking. Moreover, these findings add to the growing body of work that characterizes the multifaceted nature of children’s theories of learners^46^, by showing that learning style categories also influence their thinking. Future work should examine if and how children’s endorsements are linked to their educational choices (e.g., specialized STEM or Arts programs).

The findings more broadly build on prior work suggesting that educators and parents likely essentialize learning styles by examining the specific ways in which information about learning style traits leads to inferences about its category members^6,12^. These findings are the first to suggest that perceived learning style traits may lead parents and teachers to make a host of *specific* (unwarranted) inferences about children’s academic strengths and weaknesses. This behavior is concerning considering that learning styles are unscientifically founded seemingly arbitrary categories. It highlights that we should be wary of describing children as hands-on or visual learners as, despite any good intentions, these categories, similar to other social categories, are likely to trigger incorrect thinking about children’s abilities by educators, parents, and their peers. These findings further suggest that perceived learning styles, and the messages conveyed along with them, likely need to be accounted for in recent drives to improve the scientific foundation of early years curriculum as sensitivity to learning styles emerges early and appears to be widespread—at least in the American populations we surveyed^47^.

There are potential alternative explanations for our findings. First, people’s judgments could reflect the use of “hands-on” as a euphemism for less competent or smart. However, against this possibility are the fairly high ratings hands-on learners received in science, and the moderate ratings they received otherwise.

Second, participants may have been reflecting on real evidence-based individual differences in thinking capacities, as opposed to the VAK myth (e.g., visual/verbal capacities^48^). But, how exactly this account would explain our findings is unclear. For example, children described as visual learners were judged to be superior to children described as hands-on learners in both math and language arts, but equally skilled in science. Children described as hands-on learners were rated as being more skilled at arts, which is highly visual in nature. Put differently, visual learners’ reported strengths span subject areas that appear to vary greatly in their “visual demands”. In this way, it seems unlikely (real) individual differences in visualization capacity could accurately map onto this pattern of judgments.

Third, individuals may not spontaneously consider learning styles in their thinking about student potential in everyday life. Indeed, the experiments employed a survey method that asked participants to make, often forced-choice, decisions. This kind of method has the potential to make the effects of beliefs larger than they may be in real life because they, by their nature, require participants to pick between outcomes. However, we think it is unlikely that our choice of method alone accounts for our findings as laypeople are often encouraged to associate learning styles with educational outcomes in real-world settings. For example, many educational organizations, web sources, etc. ‘tout’ learning styles and claim that individuals with certain learning styles ‘is good at’ certain domains (e.g., kinesthetic learner—sports and arts^44,45^). Moreover, in Experiment 2, on open-ended questions, people spontaneously listed different school subjects for each learning style when it was not required.

Finally, another limitation is that our parent and teacher samples are not mutually exclusive. For example, some participants in the teacher samples may also be parents. Any potential overlap may have affected our ability to detect differences between the two groups. Regardless of the potential overlap, our findings are still informative in that they show that being a teacher ‘in and of itself’ does not protect someone from thinking that learning styles are informative predictors of learning outcomes.

A final related question is how providing descriptors or information about learning styles as opposed to only labels might have influenced the present findings. The present study is the first of its kind and so we took the most judicious approach in assigning learning styles to students. Based on prior work showing that labels promote essentialist thinking^49^, we suspect that providing labels would have only enhanced the present findings. Nonetheless, how labeling, or adjusting the descriptions, enhances or influences the present effects is an open question for future work.

Future work should investigate the consequences of the beliefs we uncovered. For example, it could be that simply labeling children as visual or hands-on learners influences others’ willingness to recommend or admit them to specialty programs, charter schools, and/or post-secondary programs. Similarly, perceived learning styles might influence children’s thinking about their own academic choices. Recent work suggests that perceived learning styles may be linked to both older children’s (i.e., approx. 10-14-years-old) and adults’ thinking about their identities (e.g., “*I’m a visual person… I would be able to pick up on math (if I was) visually solving the problem*”^50^; also in personal anecdotes^6^). In this way, it seems likely that perceived learning styles and their link to students’ identities have many overlooked effects on young students’ lives. In a related vein, future work should examine how learning style identities intersect with other identities. Learners’ identities are intersectional and include, for example, gender and racial identities among many others. Understanding intersections between these and learning style identities may be vital to building a complete picture of how young children are categorized and stereotyped in the classroom (e.g., work on brilliance^36^). Moreover, future work might also explore how the different qualities of people’s own definitions of learning styles, like the degree to which they view kinesthetic as “hands-on”, relates to their beliefs about academic aptitude.

In sum, we show that providing information about learning styles leads to (unwarranted) inferences about children’s intelligence and aptitude. They suggest more work needs to be done to characterize the impact of perceived learning styles in the classroom on children’s and teachers’ thinking and behavior.

## Methods

### Experiment 1

#### Participants

##### Children

Seventy-three 6- to 12-year children were included in the final sample (48.1% female, *M(SD)*age = 9.06(1.88) years, age range 5.67–12.83 years, 67.5% monoracial white, 49.3% female). Children came from mid to high SES families: 71.4% had an annual family income above $60,000 and 88.3% had a college-educated mother. Three additional children were tested, but excluded: 2 for not answering at least one question, and 1 for an experimenter’s error. Children were selected through a lab database of children recruited in Greensboro as well as through social media ads targeting mainly the Greensboro region. Two children were from Canada while the rest were located in the United States. Children were remunerated with an electronic activity booklet and certificate. The study was approved by the University of North Carolina Greensboro Institutional Review Board (IRB #20-0365). Parents provided written informed consent to take part in the study.

Age was treated continuously in our design; however, while recruiting we aimed to evenly recruit across the age range. The final sample included: 24 children under 8 years, 25 children between 8- and 10 years, and 24 children older than 10 years. We had hoped to collect data from 96 children but stopped testing at 73 children due to extreme difficulties with recruiting children during the pandemic and related experimenter turnover at the start of the new semester. For further information about power, including a sensitivity analysis, please see the results section.

##### Parents

Ninety-four parents were also recruited from Prolific and given $1.00 for 5 min of their time. Four additional parent participants were tested but excluded for not passing an attention check (question information provided below). All adult participants were US citizens and residents. They were 51.1% female, *M(SD)*age = 42.25(13.21), age range 20-79. The parent identity was screened using the screener question built into Prolific, “Do you have any children?” (answer “Yes”).

The data from the parent sample were collected after we collected data from the child sample. The sample size of 100 was decided upon using the significant and adequately powered effect from the child sample. Namely, using that sample as a guide, a rule of not having less than 73 in any sample after exclusions was adopted and used throughout the three studies. Further, a general target sample size of 100 per study was adopted as it is also typical in the neuromyth literature (e.g., Dekker et al., 2012; Newton & Miah, 2017). The study (along with Studies 2 and 3) was approved as exempt from further oversight by the University of North Carolina Greensboro Institutional Review Board (IRB #20-0213). All participants provided informed consent before entering the survey.

#### Materials and Procedure

##### Children

**Fig. 8:**
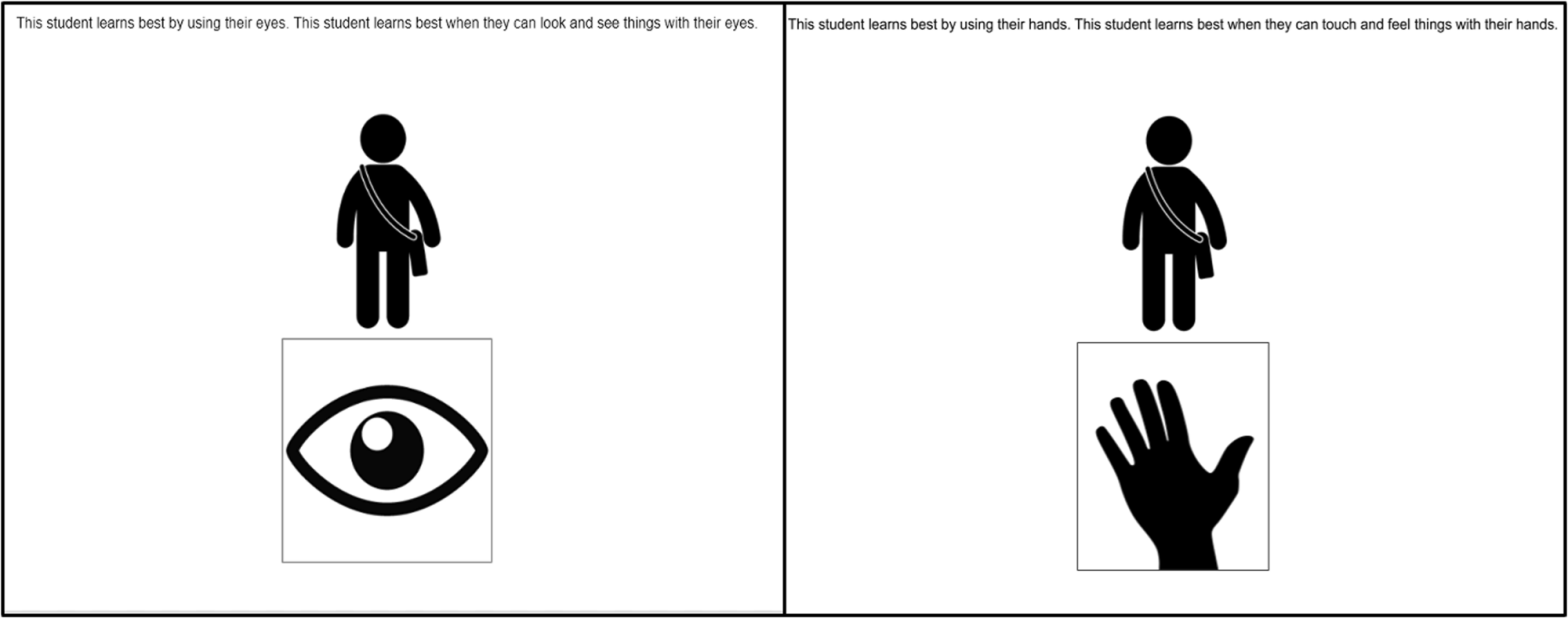
Study 1 introductions as participants saw them. Left panel: introduction to the hands-on learner. Right panel: introduction to the visual learner.

Children saw a narrated story created in PowerPoint, which was presented over a video call using either Zoom or WebEx. The story began with an introductory text explaining that the experiment was: “…going to tell you about some kids and ask you to make some guesses about them”. Children were then presented with a silhouette of a teacher leading a class and were told they were going to hear about some students and how they learn best. Children then participated in two test trials: one per learning style: hands-on and visual. In one test trial, children were told about a student who learns best using their eyes (the visual learning style), and in the other, they were told about a student who learns best by using their hands (the hands-on learning style). For example, “This student learns best by using their hands. This student learns best when they can touch and feel things with their hands”. The descriptor ‘hands-on’ was used instead of the descriptor ‘kinesthetic’ or the related descriptor ‘kinesthetic/tactile’ because we suspected the hands-on terminology would be more familiar to parents and children. Figure 8 shows how the learning style information was displayed to participants.

After each introduction, participants were asked the test questions modeled after previously published research^51^. The first questions asked about smartness, and were as follows: “Is this student smart or not very smart?” If a child answered “smart” they were then asked “Is the student sort of smart, smart, or really smart?”. Three circles increasing in size were shown under the sort of smart, smart, and really smart text. The second set of questions asked about sportiness and had the same structure (i.e., asked about whether the student was sporty and then how sporty).

The slideshow used nondescript silhouettes of people to avoid any confounding variables that may result from participants making inferences about gender or other aspects of the students’ social identity (See Fig. 8). There were four different versions of the slideshow which crossed the order of the learning styles and sporty/smart questions.

##### Parents

Parents were presented with the same stimuli as the children using Qualtrics. Parents read the stories instead of having them narrated to them. Test questions were identical. Because Qualtrics was used for survey administration versus slideshows created in PowerPoint, parents saw an entirely randomized version of the survey (i.e., the learning style information order and the question order sporty/smart were randomized). At the end of the parents’ survey, there was an attention check item asking parents to select which question was not asked in the earlier story scenario (the answer was “hands-on learners and scientific reasoning”).

### Experiment 2

#### Participants

All participants were paid $1.00 for 5 min of their time and were US citizens and residents. One hundred slots for both parents and teachers were opened on Prolific. The final samples after exclusions were 79 parent participants (46.8% female, 67.1% white) and 94 teacher participants (67.0% female, 75.5% white, *M(SD)*age = 34.94 (10.76), age range 20–78). Teachers were considered those who responded “yes” to the Prolific screening question: “You indicated that you worked in the education industry. Does your job involve teaching?”. Additional demographic information collected as part of Study 3 suggested that the majority of those who answer “yes” are teachers working in a public/private school, or university/college setting (see Study 3 for further details). Parent identity was again screened by the question, “Do you have any children?” (answer “Yes”).

Six teachers and twenty-one parents were excluded for either not answering the attention check questions in a meaningful way or not fully completing the survey. For example, in one case when asked to describe each learner some participants copied their participant ID; wrote “yes” as a description; or described the visual learner inappropriately as “deaf”. We outline the attention checks further below. Of note, exclusions were likely higher in the parent sample of this study due to an influx of new Prolific users in the later half of 2021^52^.

#### Materials and procedure

Participants filled out a survey on Qualtrics. The introductions and learning style information were presented the same as in Experiment 1. This study also included an additional attention check in the learning style introduction: After the presentation of each learner type (visual/hands-on learner), participants were asked to “Briefly describe this learner”. At test, we asked two forced-choice questions in a randomized order: (a) Which student is smarter? and (b) Which student is sportier? When answering, participants had the option to select either the hands-on or the visual learner.

An additional question at the end asked, “Do you believe that ‘individuals learn best when they receive information in their preferred learning style (e.g., visual, auditory, kinesthetic)?” to test for belief in learning styles. This question text was taken from previous research^2^. All parents and 85.1% of teachers believed in learning styles.

At the end of the survey, this study also included two open-ended exploratory questions, presented in a random order, which asked participants to list three school subjects that students with each of the two learning styles would excel at. This was done to understand what sorts of strengths teachers and parents might think those of each learning style might have. Of note, this question was not comparative in nature (e.g., “Who is better at X?”) and so it will not tell us anything about which subjects participants think a visual learner is *better at* than a hands-on learner or vice versa. Participants were excluded from the main task and this one if they did not follow instructions (i.e., they wrote something nonsensical or nothing) as such responses indicated a lack of engagement with the survey.

### Experiment 3

#### Participants

Participants were recruited from Prolific and were paid $1.00 to complete a 5-min survey. There were 100 slots opened for each sample (i.e., parent and teacher) and participants had to be US citizens and residents to be eligible. The teacher’s identity was again screened by a Prolific screening question: “You indicated that you worked in the education industry. Does your job involve teaching?” (answer “Yes”). The parents’ identity was again screened by a similar question, “Do you have any children?” (answer “Yes”). The final sample included 100 parent participants (73% female, 81% white, *M(SD)*age = 45.65 (12.23), age range 23–79) and 100 teacher participants (58% female, 66% white, *M(SD)*age = 38.76 (12.42), age range 19–75). No participants were excluded in this study as all participants passed the two attention check items which asked participants to write a sentence to describe each learner.

To gather more information about the teacher sample, teachers answered two additional questions regarding their job and work environment: “What setting do you teach in?” and “What age-group do you mainly teach?”. For the teaching setting: 57% taught in a “school (public/private)” setting, 30% in “university/college”, and the rest 13% taught in the home (e.g., home tutor) or in other settings. For the age-group: 35% taught “Adults”, 30% “High school”, 21% “Elementary school”, 11% “Middle school”, and 3% “Preschool”.

#### Materials and procedure

Participants filled out a Qualtric survey. The introductions and learning style information were presented the same as in Experiment 1 and 2. The attention check items were the same as in Experiment 2. After being introduced to the two learners in Grade 5, participants were asked to estimate grades for each learner on seven school subjects in a randomized order: math, science, language arts, social studies, art, gym, and music. Specifically, in the introductory slides they were told: “Recently, these students got their report cards. We want you to guess the grades they received on several subjects using the following 10-point scale”. Next, at test, after each learner was described, they were asked: “On the scale below make your best guess of the grades they received for each of the following subjects”. The specific 10-point letter grade scale was as follows: A + , A, A-, B + , B, B-, C + , C, C-, Below C-. These answers were converted into a numeric score for analysis: Below C- = 1, C- = 2, C = 3, C + = 4, B- = 5, B = 6, B + = 7, A- = 8, A = 9, A + = 10. The scale ranged from A+ to C- because we wanted to ensure that it was sensitive enough to detect differences among our groups. Grades below C- are rare and so we suspected that providing too many of these options (i.e., D + , D, D-, F), would lead participants to only use a narrow portion of the scale thereby reducing its sensitivity.

In efforts to replicate Experiment 2, two forced-choice questions were presented at the end of the survey in a randomized order: a) Which student is smarter? and b) Which student works harder? The smarter question was identical to Experiment 2 questions. The comparison question was changed from “sportier” to “works harder” because we were already asking about gym aptitude in the main task. For “works harder”, we predicted a similar pattern as the sportiness question wherein hands-on learners should be perceived as harder workers than visual learners.

The survey concluded with an open-ended question where we asked participants to write at least two sentences to describe what they were thinking about while they were answering the survey. This question provided a way to screen out non-attentive participants and/or bots. Again, all participants successfully passed this attention check (i.e., wrote two relevant sentences). All analyses were pre-registered.

### Reporting summary

Further information on research design is available in the Nature Research Reporting Summary linked to this article.

### Supplementary information

Supplementary Materials

Reporting Summary

## Data Availability

Data can be found at https://osf.io/exa9p/?view_only=a7e0927f83a0407a9c7bbce6fece1464.

## Code availability

Codes can be found at https://osf.io/exa9p/?view_only=a7e0927f83a0407a9c7bbce6fece1464.
