# Supplementary material for: Beware the myth: learning styles affect parents’, children’s, and teachers’ thinking about children’s academic potential
Source: NPJ Sci Learn. 2023 Oct 17;8:46. doi: 10.1038/s41539-023-00190-x (PMC10582039; doi:10.1038/s41539-023-00190-x)
Supplement: Supplementary file 1 — Supplementary Materials [file 41539_2023_190_MOESM1_ESM.docx]

***Supplementary Materials to manuscript***

**Learning styles affect parents’, children’s, and teachers’ thinking about children’s academic potential**

***Supplementary Table 1***

*Learning Styles Descriptions from Real-life Resources.*

| Visual Description | Hands-on Description | Sources (link) |
| --- | --- | --- |
| Visual learning style has a preference for **seen** or ***observed*** things, including pictures, diagrams, demonstrations, displays, handouts, films, flip-chart, etc. These people will use phrases such as ‘*show me’*, ‘let’s have a **look** at that’ and will be best able to perform a new task after reading the instructions or watching someone else do it first. These are the people who will work from lists and written directions and instructions. | Kinaesthetic learning style has a preference for *physical* experience - **touching, feeling**, ***holding,*** doing, practical **hands-on** experiences. These people will use phrases such as ‘let me try’, ‘how do you feel?’ and will be best able to perform a new task by going ahead and trying it out, learning as they go. These are the people who like to experiment, **hands-on**, and never look at the instructions first! | <https://www.trainingcoursematerial.com/free-assessment-tools/vak-learning-styles-questionnaire>  Also used in:<https://www.businessballs.com/self-awareness/vak-learning-styles/>  Also used in:  <https://americanmeditation.org/vak-learning-style-quiz/> |
| Visual: If you have this learning style then you’ll prefer **seeing** and ***observing*** things. You’ll typically work best from lists, written directions, and instructions. | Kinaesthetic: This is a practical, **hands-on** learning style. People who are kinaesthetic learners prefer the *physical* experience and often like to experiment first, rather than read the instructions. | <https://www.hfe.co.uk/learning-styles-questionnaire/> |
| Visual: a *visually-dominant* learner absorbs and retains information better when it is presented in, for example, pictures, diagrams, and charts. | Kinesthetic: a kinesthetic-dominant learner prefers a *physical* experience. She likes a **"hands-on"** approach and responds well to being able to **touch or feel** an object or learning prop. | <https://www.mindtools.com/ak6cyjn/vak-learning-styles> |
| Visual learners have two sub-channels—linguistic and spatial. Learners who are visual-linguistic like to learn through written language, such as reading and writing tasks. They remember what has been written down, even if they do not read it more than once. | Kinesthetic learners do best while **touching** and moving. It also has two sub-channels: kinesthetic (movement) and **tactile (touch).** They tend to lose concentration if there is little or no external stimulation or movement. | <http://www.nwlink.com/~donclark/hrd/styles/vakt.html> |
| If you are a visual learner, you learn by reading or **seeing** pictures. You understand and remember things by **sight**. You can picture what you are learning in your head, and you learn best by using methods that are primarily *visual.* | If you are a tactile learner, you learn by **touching** and doing. You understand and remember things through physical movement. You are a **"hands-on"** learner who prefers to **touch**, move, build, or draw what you learn, and you tend to learn better when some type of physical activity is involved. | <http://www.educationplanner.org/students/self-assessments/learning-styles-styles.shtml> |
| You process information *visually* more so than with what you hear or feel. You likely enjoy TV, a beautiful work of art, and people who are snappy dressers. You like to **see** demonstrations, pictures, diagrams, and words when learning. | You process information more by what you **feel** and experience than by what you see or hear. You likely enjoy physical activity, a sense of **touch**, exploring new things without a plan, and going with an understanding of how things make you **feel**, physically or emotionally. When learning, you are the **hands-on** type. | <https://www.proprofs.com/quiz-school/story.php?title=vak-quiz-visual-auditory-kinesthetic> |

*Note.* The search term “VAK learning style assessment” was typed into google. We examined the first page of the search results and found the following descriptions. Key terms that were also used in our descriptions are bolded, and synonyms are bolded and italicized. Words that were closely related, but were not clear synonyms were italicized. For conciseness, we included the first three sentences from longer descriptions. Please note that to get some descriptions we had to complete online quizzes.

***Supplementary Figure 1***

*Experiment 1 Line graph displaying age-related trends of ratings divided by Learning Style and Question Type.*

| 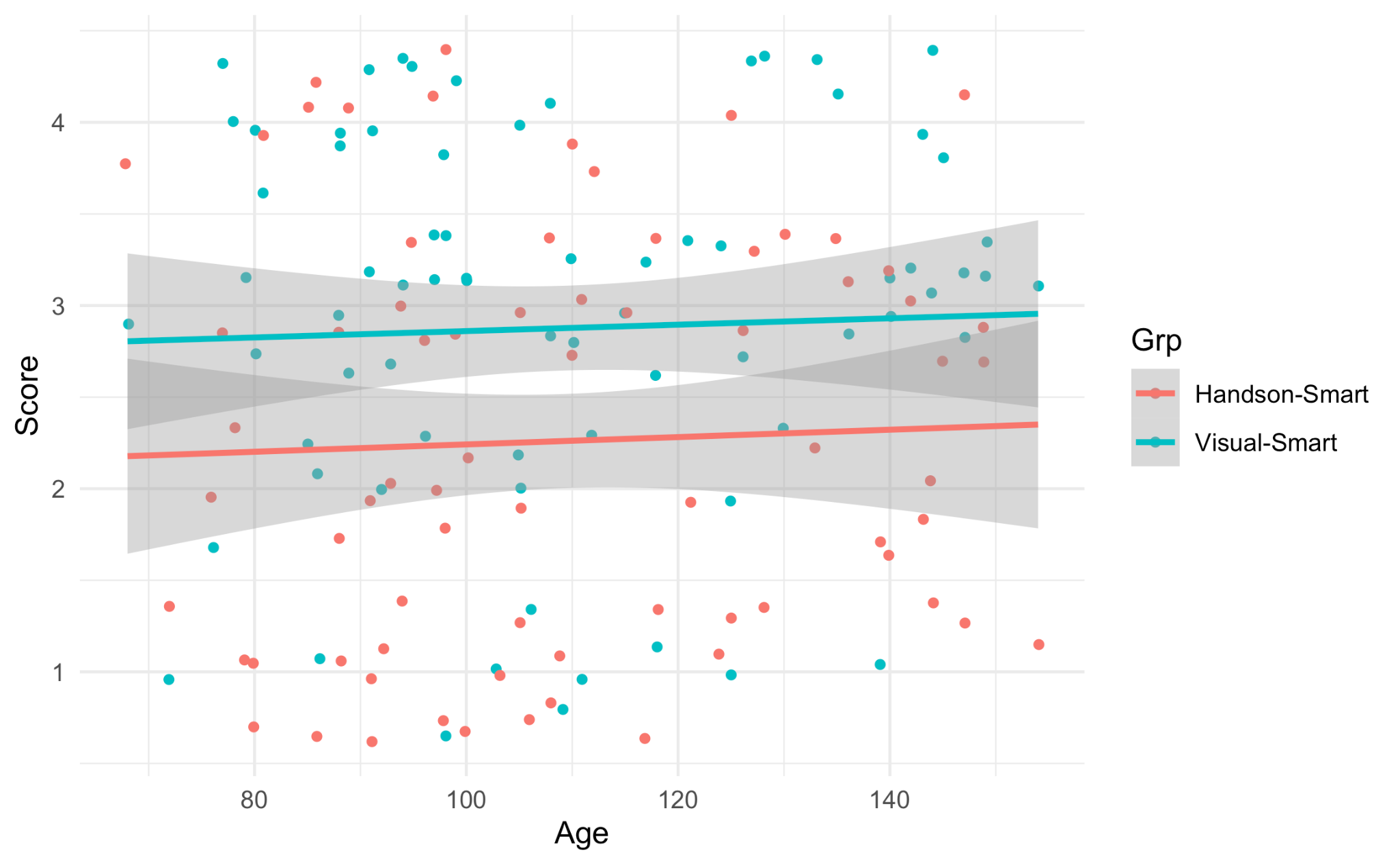   1. Rating on “smartness” | 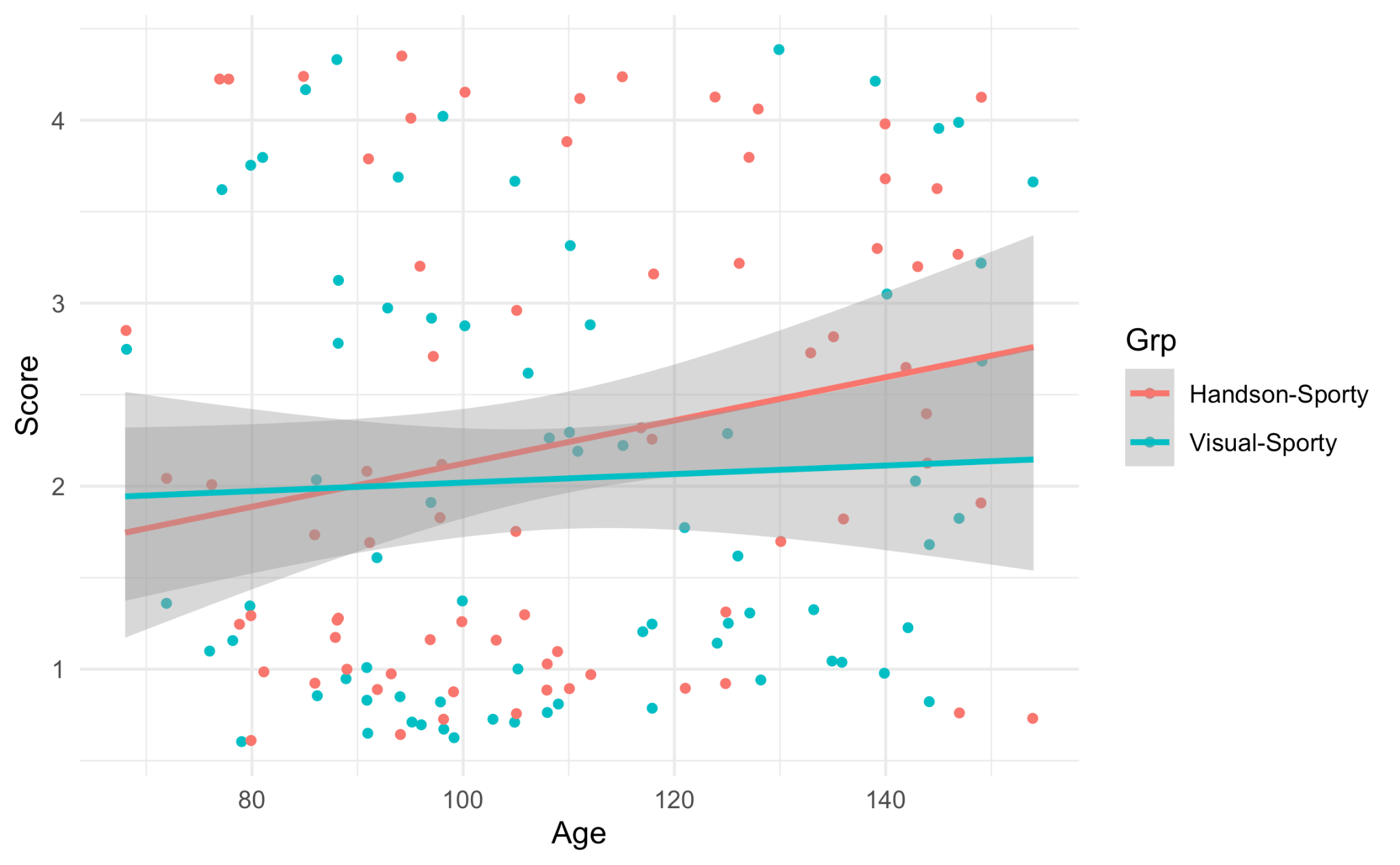   1. Rating on “sportiness” |
| --- | --- |

*Note.* Age is displayed as month to plot age continously. Points on the scatterplot are jittered to improve clarity.
