# Supplementary material for: Beware the myth: learning styles affect parents’, children’s, and teachers’ thinking about children’s academic potential
Source: NPJ Sci Learn. 2023 Oct 17;8:46. doi: 10.1038/s41539-023-00190-x (PMC10582039; doi:10.1038/s41539-023-00190-x)
Supplement: Supplementary file 2 — Reporting Summary [file 41539_2023_190_MOESM2_ESM.pdf]

nature portfolio

Corresponding author(s): Shaylene Nancekivell

Last updated by author(s): YYYY-MM-DD



# Reporting Summary

Nature Portfolio wishes to improve the reproducibility of the work that we publish. This form provides structure for consistency and transparency in reporting. For further information on Nature Portfolio policies, see our Editorial Policies and the Editorial Policy Checklist.

## Statistics

For all statistical analyses, confirm that the following items are present in the figure legend, table legend, main text, or Methods section.

| n/a | Confirmed | |
|---|---|---|
| ☐ | ☒ | The exact sample size (*n*) for each experimental group/condition, given as a discrete number and unit of measurement |
| ☐ | ☒ | A statement on whether measurements were taken from distinct samples or whether the same sample was measured repeatedly |
| ☐ | ☒ | The statistical test(s) used AND whether they are one- or two-sided<br>*Only common tests should be described solely by name; describe more complex techniques in the Methods section.* |
| ☐ | ☒ | A description of all covariates tested |
| ☐ | ☒ | A description of any assumptions or corrections, such as tests of normality and adjustment for multiple comparisons |
| ☐ | ☒ | A full description of the statistical parameters including central tendency (e.g. means) or other basic estimates (e.g. regression coefficient) AND variation (e.g. standard deviation) or associated estimates of uncertainty (e.g. confidence intervals) |
| ☐ | ☒ | For null hypothesis testing, the test statistic (e.g. *F*, *t*, *r*) with confidence intervals, effect sizes, degrees of freedom and *P* value noted<br>*Give P values as exact values whenever suitable.* |
| ☒ | ☐ | For Bayesian analysis, information on the choice of priors and Markov chain Monte Carlo settings |
| ☒ | ☐ | For hierarchical and complex designs, identification of the appropriate level for tests and full reporting of outcomes |
| ☐ | ☒ | Estimates of effect sizes (e.g. Cohen's *d*, Pearson's *r*), indicating how they were calculated |

*Our web collection on statistics for biologists contains articles on many of the points above.*

## Software and code

Policy information about availability of computer code

| | |
|---|---|
| Data collection | N/A |
| Data analysis | N/A |

For manuscripts utilizing custom algorithms or software that are central to the research but not yet described in published literature, software must be made available to editors and reviewers. We strongly encourage code deposition in a community repository (e.g. GitHub). See the Nature Portfolio guidelines for submitting code & software for further information.

## Data

Policy information about availability of data

All manuscripts must include a data availability statement. This statement should provide the following information, where applicable:

- Accession codes, unique identifiers, or web links for publicly available datasets
- A description of any restrictions on data availability
- For clinical datasets or third party data, please ensure that the statement adheres to our policy

Data and codes can be found at https://osf.io/exa9p/?view_only=a7e0927f83a0407a9c7bbce6fece1464.

## Research involving human participants, their data, or biological material

Policy information about studies with human participants or human data. See also policy information about sex, gender (identity/presentation), and sexual orientation and race, ethnicity and racism.

| | |
|---|---|
| Reporting on sex and gender | We followed the sex, gender (identity/presentation) and sexual orientation policy to describe our participants. |
| Reporting on race, ethnicity, or other socially relevant groupings | We followed the race, ethnicity and racism policy to describe our participants. |
| Population characteristics | We used age as a covariate for the Study 1 child data analysis. |
| Recruitment | Study 1 children were selected through a lab database of children recruited in the Greensboro as well as through social media ads targeting mainly the Greensboro region. All adult samples were recruited from Prolific. |
| Ethics oversight | Study 1 child portion was approved by the University of North Carolina Greensboro Institutional Review Board (IRB #20-0365). Parents provided written informed consent to take part in the study. All adult data collection was approved as exempt from further oversight by the University of North Carolina Greensboro Institutional Review Board (IRB #20-0213). All participants provided informed consent before entering the survey. |

Note that full information on the approval of the study protocol must also be provided in the manuscript.

# Field-specific reporting

Please select the one below that is the best fit for your research. If you are not sure, read the appropriate sections before making your selection.

☐ Life sciences ☒ Behavioural & social sciences ☐ Ecological, evolutionary & environmental sciences

For a reference copy of the document with all sections, see nature.com/documents/nr-reporting-summary-flat.pdf

# Behavioural & social sciences study design

All studies must disclose on these points even when the disclosure is negative.

| | |
|---|---|
| Study description | Studies 1-3 all used quantitative methods. |
| Research sample | Study 1 included child and parent samples, Studies 2 and 3 included parent and teacher samples. All adult samples were collected online from Prolific, and the child data was collected from local regions in North Carolina, USA (middle class). The samples are from North America and are mostly white and from middle class. |
| Sampling strategy | Studies 1-3 used convenient samples. Data from the adult samples replicated each other across studies. In terms of sample sizes, we referred to previously published papers with similar topics. |
| Data collection | Child data were collected with an experimenter conducting an 1-on-1 interview. Adult participants complete the questionnaire online anonymously using their own devices. |
| Timing | Child data: 2020 spring-fall; Adult data: 2020 summer-fall. |
| Data exclusions | Data was excluded if children was not able to answer a question, and if adults failed to pass the attention check items. |
| Non-participation | No known dropouts. |
| Randomization | Questions/scenarios were randomly presented across participants. |

# Reporting for specific materials, systems and methods

We require information from authors about some types of materials, experimental systems and methods used in many studies. Here, indicate whether each material, system or method listed is relevant to your study. If you are not sure if a list item applies to your research, read the appropriate section before selecting a response.

## Materials & experimental systems

| n/a | Involved in the study |
| --- | --- |
| ☒ | ☐ Antibodies |
| ☒ | ☐ Eukaryotic cell lines |
| ☒ | ☐ Palaeontology and archaeology |
| ☒ | ☐ Animals and other organisms |
| ☒ | ☐ Clinical data |
| ☒ | ☐ Dual use research of concern |
| ☒ | ☐ Plants |

## Methods

| n/a | Involved in the study |
| --- | --- |
| ☒ | ☐ ChIP-seq |
| ☒ | ☐ Flow cytometry |
| ☒ | ☐ MRI-based neuroimaging |
